# Isoniazid urine spectrophotometry for prediction of serum pharmacokinetics in adults with TB

**DOI:** 10.5588/ijtldopen.23.0361

**Published:** 2024-02-01

**Authors:** P. S. Rao, K. Reed, N. Modi, D. Handler, K. Petros de Guex, S. Yu, L. Kagan, R. Reiss, N. Narayanan, C. A. Peloquin, A. Lardizabal, C. Vinnard, T. A. Thomas, Y. L. Xie, S. K. Heysell

**Affiliations:** ^1^ Division of Infectious Diseases and International Health, and; ^2^ School of Arts and Sciences, University of Virginia, Charlottesville, VA,; ^3^ Public Health Research Institute and Global Tuberculosis Institute, Rutgers New Jersey Medical School, Newark, NJ,; ^4^ Department of Pharmaceutics and Center of Excellence for Pharmaceutical Translational Research and Education, Ernest Mario School of Pharmacy, Rutgers State University of New Jersey, Newark, NJ,; ^5^ College of Pharmacy and Emerging Pathogens Institute, University of Florida, Gainesville, FL, USA

**Keywords:** tuberculosis, INH, LC-MS/MS, PK

## Abstract

**BACKGROUND:**

Isoniazid (INH) is an important drug in many TB regimens, and unfavorable treatment outcomes can be caused by suboptimal pharmacokinetics. Dose adjustment can be personalized by measuring peak serum concentrations; however, the process involves cold-chain preservation and laboratory techniques such as liquid chromatography (LC)/mass spectrometry (MS), which are unavailable in many high-burden settings. Urine spectrophotometry could provide a low-cost alternative with simple sampling and quantification methods.

**METHODS:**

We enrolled 56 adult patients on treatment for active TB. Serum was collected at 0, 1, 2, 4, 6, and 8 h for measurement of INH concentrations using validated LC-MS/MS methods. Urine was collected at 0–4, 4–8, and 8–24 h intervals, with INH concentrations measured using colorimetric methods.

**RESULTS:**

The median peak serum concentration and total serum exposure over 24 h were 4.8 mg/L and 16.4 mg*hour/L, respectively. Area under the receiver operator characteristic curves for urine values predicting a subtherapeutic serum concentration (peak <3.0 mg/L) were as follows: 0–4 h interval (AUC 0.85, 95% CI 0.7–0.96), 0–8 h interval (AUC 0.85, 95% CI 0.71–0.96), and 0–24 h urine collection interval (AUC 0.84, 95% CI 0.68–0.96).

**CONCLUSION:**

Urine spectrophotometry may improve feasibility of personalized dosing in high TB burden regions but requires further study of target attainment following dose adjustment based on a urine threshold.

## RÉSUMÉ

**CONTEXTE:**

L'isoniazide (INH) est un médicament important dans de nombreux schémas thérapeutiques contre la TB, et des résultats thérapeutiques défavorables peuvent être dus à une pharmacocinétique sous-optimale. L'ajustement de la dose peut être personnalisé en mesurant les concentrations sériques maximales ; cependant, le processus implique la conservation de la chaîne du froid et des techniques de laboratoire telles que la chromatographie liquide (LC)/spectrométrie de masse (MS), qui ne sont pas disponibles dans de nombreuses régions à forte charge de morbidité. La spectrophotométrie urinaire pourrait constituer une alternative peu coûteuse avec des méthodes d'échantillonnage et de quantification simples.

**MÉTHODES:**

Nous avons recruté 56 patients adultes sous traitement pour une TB active. Le sérum a été prélevé à 0, 1, 2, 4, 6 et 8 h pour mesurer les concentrations d'INH à l'aide de méthodes LC-MS/MS validées. L'urine a été prélevée à des intervalles de 0–4, 4–8 et 8–24 h, et les concentrations d'INH ont été mesurées à l'aide de méthodes colorimétriques.

**RÉSULTATS:**

La concentration sérique maximale médiane et l'exposition sérique totale sur 24 h étaient respectivement de 4,8 mg/L et de 16,4 mg*heure/L. L'aire sous les courbes caractéristiques de l'opérateur récepteur a été mesurée à l'aide de méthodes colorimétriques. Les aires sous les courbes caractéristiques des récepteurs pour les valeurs urinaires prédisant une concentration sérique sous-thérapeutique (pic <3,0 mg/L) étaient les suivantes : intervalle 0–4 h (AUC 0,85 ; IC 95% 0,7–0,96), intervalle 0–8 h (AUC 0,85 ; IC 95% 0,71–0,96), et intervalle de collecte d'urine 0–24 h (AUC 0,84 ; IC 95% 0,68–0,96).

**CONCLUSION:**

La spectrophotométrie urinaire peut améliorer la faisabilité d'un dosage personnalisé dans les régions à forte charge de TB, mais nécessite une étude plus approfondie de l'atteinte de la cible après l'ajustement de la dose sur la base d'un seuil urinaire.

Isoniazid (INH) is an important first-line medication in treating active and latent TB, including at high dose in some regimens for rifampin (RIF) resistant TB.^1,2^ INH rapidly reduces bacillary load,^3^ and low drug concentrations have been associated with treatment failures.^4,5^ Pharmacokinetic (PK) variability of INH is an important contributor to unfavorable treatment outcomes, including acquired drug resistance.^6^ Data reported from 156 countries between 2002 and 2018 indicate that 7.4% of newly diagnosed TB patients and 11.4% of previous treated patients had INH resistance.^7^ Typical daily doses are 300 mg or roughly 5 mg/kg for active TB treatment and the peak plasma/serum concentrations (C_max_) range from 3 mg/L to 6 mg/L.^8^

The total serum exposure or area under the concentration curve over 24 h (AUC_0–24_, mg*hour/L) is studied as a predictor of clinical outcomes, and an INH serum AUC_0–24_ of 52 mg*hour/L^9^ and an AUC_0–24_/minimum inhibitory concentration (MIC) ratio of 567^10,11^ have been established in preclinical models and consensus expert opinion as important thresholds for bacterial kill and prevention of resistance.^12,13^ However, the multiple blood draws throughout the dosing interval necessary for AUC_0–24_ calculations, including logistical challenges of cold-chain preservation for blood draws in certain environments and the lack of availability or standardization of MIC testing of an individual’s infecting *Mycobacterium tuberculosis* isolate in most laboratories, has limited the use of PK data to personalize a dose and serum exposure for the majority of people treated for TB globally. Consequently, C_max_ has been used as the common pragmatic target for therapeutic drug monitoring in clinical settings.^14^

In the few clinical settings where serum exposure-based dose adjustment for INH is performed, a limited sampling strategy of blood draws is often used to estimate C_max_, and dose is increased if a minimum target of 3 mg/L is not reached.^15,16^ This strategy allows venous phlebotomy in windows of time fitting for a clinic or home visit, but still requires centrifugation, and transfer or shipment of the frozen plasma, or serum to a central laboratory with validated high-performance liquid chromatography (LC) or mass spectrometry (MS) capabilities.

Alternative sampling methods such as using dry blood spots, urine, saliva, or hair, all offer potential solutions to serum sampling for many anti-TB medications, but each method has unique limitations.^17^ Urine has been used for over 50 years to monitor treatment adherence to INH, but most studies have used qualitative detection methods.^18–20^ We have demonstrated the quantification of other anti-TB medications in different patient populations using a low-cost spectrophotometer with laboratory methods available in most clinical settings.^21–23^

In this prospective study, we used spectrophotometric methods to quantify the amount of INH eliminated in urine, and sought to determine the urine collection interval that would be most indicative of serum exposures relevant for dose adjustment in routine care.

## METHODS

### Study participants

In order to compare a urine spectrophotometric quantification or INH excretion with the gold standard serum concentration and PK parameters used for dose adjustment, adults newly diagnosed with pulmonary TB were enrolled through health departments across Virginia and New Jersey, USA. Patients were enrolled during the intensive phase of treatment while taking daily first-line treatment, most commonly RIF, INH, pyrazinamide, and ethambutol, or in the continuation phase of treatment taking daily RIF and INH. The enrollment period spanned 3.5 years, with a target of at least 60 people taking RIF (findings reported separately),^24^ and 50 people taking INH to evaluate pre-specified covariates.

All eligible participants signed informed consent for the protocol approved at Rutgers Health Sciences, Newark, NJ, USA (IRB Pro2018001857); and University of Virginia Health Sciences, Charlottesville, VA, USA (IRB HSR #20944). The findings were reported per STARD (Standards for Reporting of Diagnostic Accuracy) guidelines when able for prospective diagnostic accuracy studies.^25^

### Study procedures

The PK visit was conducted at least 2 weeks after medication initiation to allow for steady state kinetics. Participants were administered anti-TB medications using directly observed therapy, including on the PK visit. Pre-dose venous blood was drawn. Upon medication ingestion, blood was collected at 1, 2, 4, 6, and 8 h post-dose. Blood samples were centrifuged, and serum was frozen at –80^°^C until batch shipment to University of Florida, Gainesville, FL, USA, for serum analysis using validated LC-MS/MS methods.^26^ Similarly, a baseline urine sample was collected before medication administration and post-dose urine was collected at the 0–4 h, 4–8 h, and 8–24 h time intervals. Pooling was done to obtain 0–8 h and 0–24 h urine to determine total drug excretion over 24 h. Urine collection volumes were recorded, and aliquots made for every time interval were frozen at –80^°^C until analysis.

### Urine assay

The steps to measure INH in urine in a spectrophotometer using colorimetric principles are described. The method followed was adapted from Boxenbaum et al. and was used to quantify total INH in urine.^27^ In brief, for a working volume of 500 µL of urine, 50 µL of hydrochloric acid was added, along with 83 µL of sodium acetate, 800 µL of isobutanol, and 50 µL of ammonium sulfate. The mixture was centrifuged at 13,000 rpm for 5 min, and 500 µL of the top layer was transferred to a fresh vial. To this vial, 83 µL of sodium acetate and 500 µL of ethyl acetate–heptane mixture was added and centrifuged at 13,000 rpm for 5 min; 500 µL of the top layer formed was once again transferred to another fresh vial, where 200 µL of hydrochloric acid and 7.5 µL of vanillin–ethanol mixture were added. From the total mixture of 707.5 µl, 100 µL of the aqueous phase was added to a 96-well plate and the absorbance was measured at 380 nm in a BioTek Synergy H4 Hybrid Reader (Agilent, Fisher Scientific, Göteborg, Sweden). A calibration curve was constructed with concentrations of 1,000, 500, 250, 125, 62.5, and 31.25 mg/L.

### Statistical analysis

PK parameters were determined by non-compartmental analysis using Phoenix WinNonlin v8.3 (Certara, Princeton, NJ, USA). C_max_ was the maximum concentration measured, and total exposure over 24 h (AUC_0–24_) was estimated using concentrations in the elimination phase. INH dosing was not uniform across all participants due to weight-based dosing, and INH in urine was normalized by converting INH amounts to percentage dose excreted. Spearman’s correlations were performed between percentage INH eliminated in urine at each collection interval and serum C_max_ and AUC_0–24_. Receiver operating characteristic (ROC) curves were constructed for urine at various time intervals to predict a corresponding minimum serum C_max_ target <3.0 mg/L. Descriptive statistics were used to characterize demographics, Mann–Whitney *U*-tests to compare covariates, and concentration–time profile scatter diagram was plotted for all participants. Demographic data and correlations were performed in MS Excel (Microsoft, Redmond, WA, USA); and ROC analysis was performed in R software v3.6.1 (R Computing, Vienna, Austria; http://r-project.org) using the *pROC* package.^28^

## RESULTS

**Figure 1.**
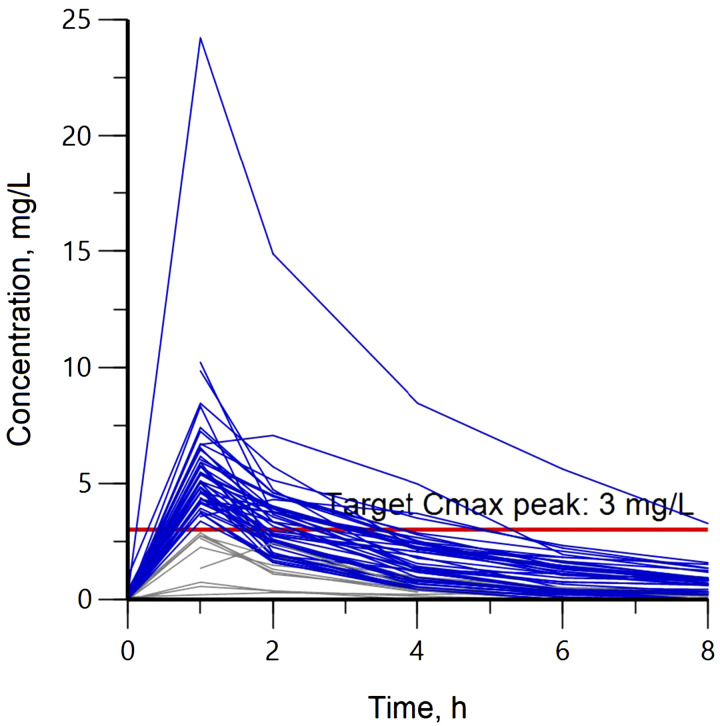
Individual serum concentration-time profile for isoniazid. Blue lines represent participants who reached C_max_ over 3.0 mg/L and gray lines depict participants that did not attain the target concentration. The horizontal solid-red line indicates the target C_max_ peak of 3.0 mg/L. C_max_ = peak serum concentration.

We enrolled 60 adults across the two sites, and 56 of the participants were taking INH as part of their multi-drug regimen. The median age was 45.5 years, and there were 20 females. Fourteen participants had diabetes and one person was living with HIV. Other demographic characteristics are presented in Table 1. INH concentration–time profiles for all 56 participants are shown in Figure 1. Forty-seven (84%) reached serum target of 3.0 mg/L, with a median C_max_ of 4.8 mg/L. The median AUC_0–24_ and weight-normalized dose were 16.4 mg*hour/L and 4.6 mg/kg, respectively, with only one participant (2%) reaching an AUC_0–24_ target of 52 mg*hour/L. Other PK parameters are presented in Table 2.

**Table 1.** Demographic characteristics at pharmacokinetic study visit.

	(*n* = 56)
Parameter	*n* (%)
Age, years, median [IQR]	45.5 [32–54]
Female	20 (35.7)
Weight, kg, median [IQR]	66.6 [56.7–73.8]
BMI, kg/m^2^, median [IQR]	23.7 [20.7–25.2]
Diabetes	14 (25)
HIV	1 (1.7)

IQR = interquartile range; BMI = body mass index.

**Table 2.** Pharmacokinetic parameters of isoniazid.

Parameter	Median [IQR]
Isoniazid dose, mg	300 [300–300]*
Weight-adjusted dosage, mg/kg	4.6 [4.1–5.3]
C_max_, mg/L	4.8 [3.9–5.9]
T_max_, h	1 [1.0–1.0]
AUC_0–24_, mg*hour/L	16.4 [9.7–22.8]
t_1/2_, h	2.2 [1.2–2.8]
Oral volume of distribution, L	57.6 [46.5–69.9]
Oral clearance, L/h	20.8 [13.2–31.9]

* Range of isoniazid dose: 300–600 mg.

C_max_ = peak serum concentration; T_max_ = time to peak concentration; AUC_0–24_ = area under the concentration curve over 24 h; t_1/2_ = half-life.

### Urine colorimetry

**Figure 2.**
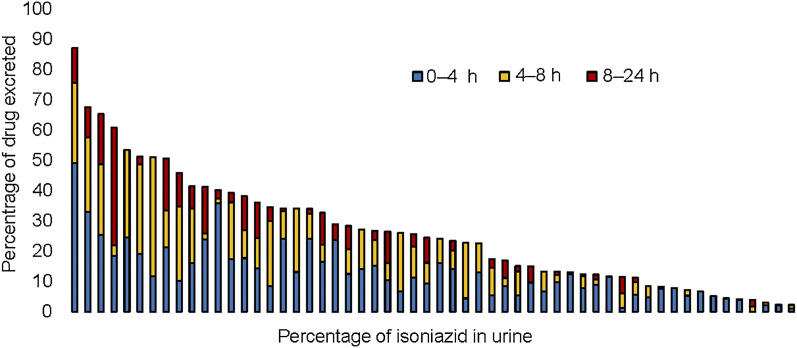
Percentage of isoniazid dose excreted in urine over 0–4 h, 4–8 h, and 8–24 h collection intervals with each bar representing an individual participant’s measured percentage of isoniazid dose excreted over 24 h.

Colorimetric analysis demonstrated a linear relationship for INH concentrations between 31.2 mg/L and 1,000 mg/L (*r*^2^ = 0.99). The highest percentage of INH excreted over 24 h was 87.1%, and the lowest was 2.3% of the total dose consumed. The percentage of INH excreted in urine during 0–4 h, 4–8 h, and 8–24 h collection intervals are presented in Figure 2, with a pattern of the greatest proportion of dose excreted occurring in the first 8 h.

Spearman’s rank correlation coefficient (ρ) of urine excretion was highest at 0–8 h pooled collection interval for both serum C_max_ (ρ = 0.36, *P* < 0.001) and AUC_0–24_ (ρ = 0.72, *P* < 0.001). Correlation coefficients at other urine collection times are provided in Supplementary Table S1. Mann-Whitney *U*-tests did not find any differences in the proportions of INH excreted in urine among the covariates sex, diabetes, and HIV status.

**Figure 3.**
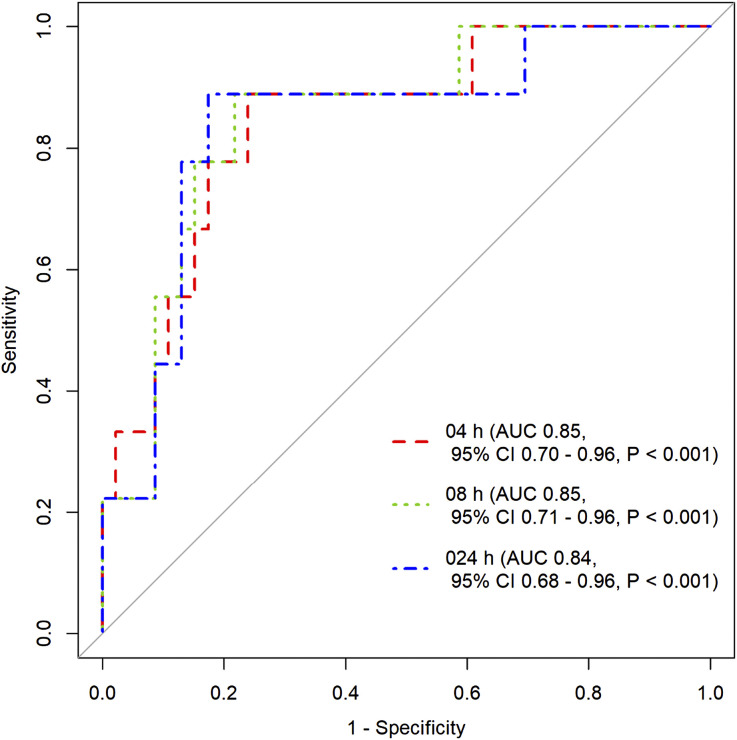
Receiver operating characteristic curves to determine C_max_ <3.0 mg/L at 0–4 h, 4–8 h, 8–24 h urine collection intervals. AUC = area under the curve: CI = confidence interval; C_max_ = peak serum concentration.

ROC curves were generated for INH in urine and C_max_ to predict concentrations below the minimum target of 3.0 mg/L commonly used for dose adjustment.^14^ Area under the ROC curve (AUC of ROC) was above 0.8 for three intervals of urinary excretion that included the first 0–4 h collection interval: 0–4 h interval: AUC 0.85, 95% confidence interval [CI] 0.7–0.96; 0–8 h interval: AUC 0.85, 95% CI 0.71–0.96; and 0–24 h urine collection interval: AUC 0.84, 95% CI 0.68–0.96 (Figure 3). AUC for ROC curves for other urine collection intervals are presented in Supplementary Table S2. Sensitivity and specificity thresholds were calculated; for a minimum sensitivity of 80%, the assay was 78% specific, and for a minimum specificity of 80% the assay was 77% sensitive for predicting subtarget serum C_max_ exposures from the 0–8 h urine interval. Other sensitivities and specificities are listed in Supplementary Table S2.

## DISCUSSION

We were able to measure INH concentrations in urine using a spectrophotometer at different collection intervals over 24 h that correlated well with serum concentrations. Urinary excretion was particularly useful for predicting underexposure and serum C_max_ below the minimum target typically used for INH dose increase in clinical settings. Given similar performance across all urine collection intervals, a 0–4 h time period of urine collection may be most practical for further clinical evaluation.

The highest correlation between urine and maximum serum concentration for urine collection intervals over 24 h was obtained within 8 h of medication administration and the correlation coefficient was lowest (ρ = 0.14, *P* = 0.76; Supplementary Table S1) for the 8–24 h urine interval when most of the dose had already been eliminated.

While our primary hypothesis was that urinary excretion could predict individuals with subtarget INH C_max_, we also found a strong correlation between the total serum exposure, AUC_0–24_, and 0–8 h pooled urine (*r* = 0.72, *P* < 0.001; Supplementary Table S1). The clinical practice of dose adjustment using a C_max_ target is a proxy for optimizing total exposure (AUC_0–24_) and used in the majority of settings where multiple blood draws throughout a dosing interval is impractical. To note, despite the majority of patients in our study reaching a minimum serum C_max_ target, the median AUC_0–24_ of 16.4 mg*hour/L was well below targets such as 52 mg*hour/L found in at least one prospective study of patients with active pulmonary TB as predictive of long-term treatment outcomes.^6,8,9^ Further study should explore urinary excretion-based INH dose adjustment to reach higher AUC_0–24_ targets.

It is logistically appealing to examine if a ‘spot’ pre-dose urine sample could determine if any residual INH detection from the prior dose predicted those with above target serum exposure.^19,29^ However, in the 50 patients for which a pre-dose urine sample was collected, INH was not detected in 13, and the remaining had values below the limit of the assay’s detection. Sophisticated detection methods such as LC or MS might be able to quantify low pre-dose INH concentrations, but this would require sending samples to tertiary laboratories with instrument capabilities. More sensitive spectrophotometric methods need to be optimized for a wider limit of drug detection in urine.

There were limitations to this study on diagnostic accuracy. Given that interindividual variability plays a significant role in INH PK,^6,8,30,31^ and was also evident in our cohort, measurement of all major determinants of PK variability may have refined understanding of the urine assay’s performance. While Mann–Whitney *U*-tests did not find differences in urinary assay correlation with serum targets based on sex or presence of diabetes, there was only one patient living with HIV and only three patients with reduced creatinine clearance below 60 mg/dL, which limited comparisons of those two covariates. It should be noted that the *NAT2* genotype, and the consequent INH acetylator phenotype, were not available for comparison to the urine spectrophotometry assay for prediction of serum INH targets. As *NAT2* genotyping moves closer to the point-of-care such as has been demonstrated with whole blood and the GeneXpert platform (Cepheid, Sunnyvale, CA, USA),^32^ there may be a role for initial screening using *NAT2* polymorphisms, with subsequent triage of fast acetylators to urinary excretion testing for personalized dose adjustment, and the yield of such an algorithm could be tested in comparison to dose adjustment based on *NAT2* genotype alone.^33,34^

We examined the ROC curves for the different urinary collection intervals, and also included a 2 h serum value in comparison for predicting the serum C_max_ minimum target, given that time point for blood collection is commonly used as means for capturing near-peak concentrations of INH, RIF, ethambutol, and pyrazinamide for patients on a common intensive phase regimen for drug-susceptible TB.^14^ However, all but one patient in our cohort reached INH C_max_ at 1 h post-dose, and thus in settings where serum drug quantification is available, this collection time would be clearly preferred. Furthermore, while testing of urinary excretion using colorimetric methods reduces the need for sophisticated instruments and trained personnel, it does not entirely eliminate the requirement of laboratory facilities for centrifugation, chemical storage/mixing, and spectrophotometry with ultraviolet capabilities. Further development should therefore prioritize optimization of the urine sample processing steps.

In summary, spectrophotometry for the quantification of urinary INH excretion predicted clinically significant serum values used for dose adjustment. A 4-h urine collection after observed dose is fitting for a planned clinic or home visit and local laboratory processing. Future study of serum target attainment based on dose adjustment from INH dose adjustment may considerably expand access to personalized care for people with TB across a variety of clinical environments.

## Supplementary Material


