# Supplementary material for: Isoniazid urine spectrophotometry for prediction of serum pharmacokinetics in adults with TB
Source: IJTLD Open. Author manuscript; Available in PMC 2024 Apr 23. (PMC11037464; doi:10.5588/ijtldopen.23.0361)
Supplement: Supplementary file [file NIHMS1984854-supplement-Supplementary_file.pdf]

©2024 The Union http://dx.doi.org/10.5588/ijtldopen.23.0361

Isoniazid urine spectrophotometry for prediction of serum pharmacokinetics in adults with TB

Supplementary Table S1: Correlation coefficients for 2-hour serum isoniazid (single time point used clinically for dose adjustment) and urine isoniazid excretion at various collection intervals with maximum isoniazid serum concentration (peak-Cmax) and total exposures (AUC0-24).

	Correlation coefficient ρ (p-value)	
Dependent variable	Cmax	AUC0-24
2-hour serum	0.71 (p<0.001)	0.93 (p<0.001)
0-4 hour urine	0.34 (p=0.01)	0.56 (p<0.001)
4-8 hour urine	0.25 (p=0.06)	0.63 (p<0.001)
0-8 hour urine	0.36 (p=0.007)	0.72 (p<0.001)
8-24 hour urine	0.04 (p=0.76)	0.17 (p=0.2)
0-24 hour urine	0.32 (p=0.01)	0.65 (p<0.001)

Supplementary Table S2: Area under the receiver operating characteristic curve (AUC) along with 95% confidence intervals (95% CI), sensitivity, and specificity for 2-hour serum concentration (single time point used clinically for dose adjustment) and amount of isoniazid excreted in urine at various collection intervals for a maximum serum (peak-Cmax) threshold of 3.0 mg/L.

Dependent variable	ROC for Cmax target for dose adjustment		
	AUC (95% CI)	Sensitivity at 80% specificity	Specificity at 80% sensitivity
2 hour serum	0.95 (0.89-1.0)	88%	87%
0-4 hour urine	0.84 (0.71-0.98)	77%	76%
4-8 hour urine	0.68 (0.53-0.83)	22%	64%
0-8 hour urine	0.85 (0.71-0.98)	77%	78%
8-24 hour urine	0.63 (0.43-0.82)	0%	32%
0-24 hour urine	0.84 (0.68-0.99)	88%	82%
